# Corrections to “Effects of Targeted Assistance and Perturbations on the Relationship Between Pelvis Motion and Step Width in People With Chronic Stroke”

**DOI:** 10.1109/TNSRE.2025.3563816

**Published:** 2025

**Authors:** Nicholas K. Reimold, Holly A. Knapp, Alyssa N. Chesnutt, Alexa Agne, Jesse C. Dean

**Affiliations:** Department of Health Professions, Medical University of South Carolina, Charleston, SC 29425 USA; Department of Health Professions, Medical University of South Carolina, Charleston, SC 29425 USA; Department of Health Professions, Medical University of South Carolina, Charleston, SC 29425 USA; Department of Health Professions, Medical University of South Carolina, Charleston, SC 29425 USA; Department of Health Professions, Medical University of South Carolina, Charleston, SC 29425 USA, and also with Ralph H. Johnson VAMC, Charleston, SC 29401 USA

IN THE above article [1], Figure 5 is incorrectly presented as a duplicate of Figure 4. The correct figure is presented here.

## Figures and Tables

**Fig. 5.**
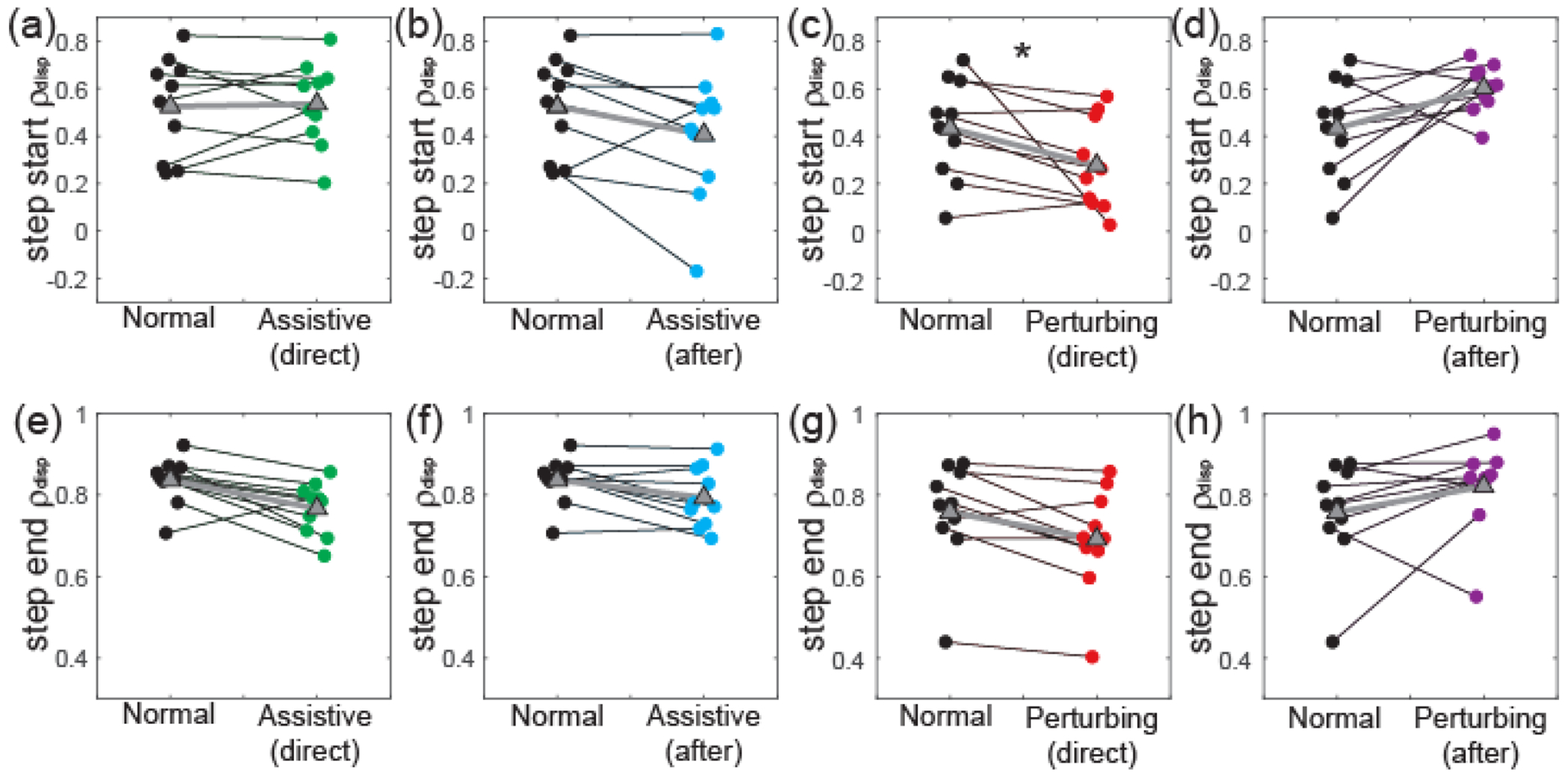
Force-field effects on non-paretic *ρ*_disp_. The figure structure matches that for Figure 4.
