# Supplementary material for: Effects of Targeted Assistance and Perturbations on the Relationship Between Pelvis Motion and Step Width in People With Chronic Stroke
Source: IEEE Trans Neural Syst Rehabil Eng. Author manuscript; Available in PMC 2022 Feb 15. (PMC8844911; doi:10.1109/TNSRE.2020.3038173)
Supplement: Supplementary Material [file NIHMS1777295-supplement-Supplementary_Material.pdf]

Supplementary Material for: "Effects of targeted assistance and perturbations on the relationship between pelvis motion and step width in people with chronic stroke"

APPENDIX A

I. INTRODUCTION

People with chronic stroke (PwCS) often exhibit a weaker relationship between mediolateral pelvis displacement and step width for steps taken with their paretic leg [1]. As mechanics-dependent adjustments in step width are an important gait stabilization strategy [2], this weaker relationship may contribute to deficits in post-stroke balance. To investigate this topic, we developed a force-field able to encourage targeted step widths on a step-by-step basis [3].

In neurologically-intact controls, several biomechanical factors predict the step width for a given step, as influenced by both the body's passive dynamic properties and active control [4]. Most prominently, people tend to place their swing leg more laterally to produce wider steps when the pelvis is displaced farther mediolaterally from the stance foot [5-6]. While not as strong a relationship, wider steps also tend to accompany larger mediolateral velocities away from the stance foot [5-6]. Additionally, these step-by-step fluctuations vary around a mean step width value that differs between individuals – likely due in part to differences in anthropometric factors such as pelvis width or leg circumference [7], and possibly differences in an individuals' balance confidence [8].

Our prior work has investigated the effects of an assistive force-field that used various combinations of these three factors (pelvis displacement, pelvis velocity, mean step width) to predict mechanically-appropriate step widths in neurologically-intact participants [9]. All force-field control equations included mediolateral pelvis displacement, and all equations significantly increased our primary outcome measure quantifying the relationship between pelvis displacement and step width – step start ρ_{disp}. These increases were significantly larger for equations that also included pelvis velocity, while the inclusion of mean step width appeared not to play an important role. However, it is unclear whether similar effects would be observed in PwCS, a population in whom step start ρ_{disp} is likely reduced for steps taken with the paretic leg.

The purpose of this pilot experiment was to determine whether the relationship between pelvis displacement and paretic step width was differently affected by several candidate equations used to control force-field assistance. Specifically, we investigated the direct effects of equations that included various combinations of pelvis displacement, pelvis velocity, and the individual participant's mean step width. This experiment was not hypothesis-driven, but was used to identify the force-field equation to be used in our main experiment.

II. METHODS

A. Participants

Twelve PwCS completed this study. Basic participant demographic information is provided in Table S1. The inclusion and exclusion criteria match those presented in the main text, with the exception that we did not have a maximum step start paretic ρ_{disp} value for this preliminary experiment. All participants provided informed consent using a form approved by the Medical University of South Carolina Institutional Review Board and consistent with the Declaration of Helsinki.

B. Experimental Protocol

Participants performed a series of 2-minute walking trials at their normal walking speed, identified as described in the main text. For all trials, participants wore a harness to prevent falls and were not permitted to hold onto a handrail. For the first (Normal) treadmill trial, participants did not interact with the force field. Participants then performed four randomized-order walking trials in which assistance was controlled based on the four equations detailed below. The equations differed in terms of which combination of factors (from pelvis displacement, pelvis velocity, and mean step width) were used to predict a mechanically-appropriate step width. These trials were separated by 2-minute wash-out periods in which participants walked with the force-field in Transparent mode. The present

TABLE SI
PARTICIPANT CHARACTERISTICS

#	Age (yr)	Time since stroke (mo)	Paretic side	Gender	LEFM score	Walking speed (m/s)
1	53	19	L	F	24	0.30
2	23	74	R	M	24	0.80
3	71	39	R	M	23	0.40
4	82	27	R	F	26	0.25
5	33	103	L	F	21	1.20
6	38	80	L	M	15	0.45
7	71	59	R	M	30	0.30
8	76	13	R	F	34	0.70
9	54	26	L	F	24	0.70
10	56	201	R	F	34	0.55
11	52	36	L	F	24	0.60
12	76	25	R	M	25	0.30
group	57±19	59±53	5L/7R	7F/5M	25±5	0.55±0.28

Participant demographic characteristics, clinical measures, and treadmill walking speeds.

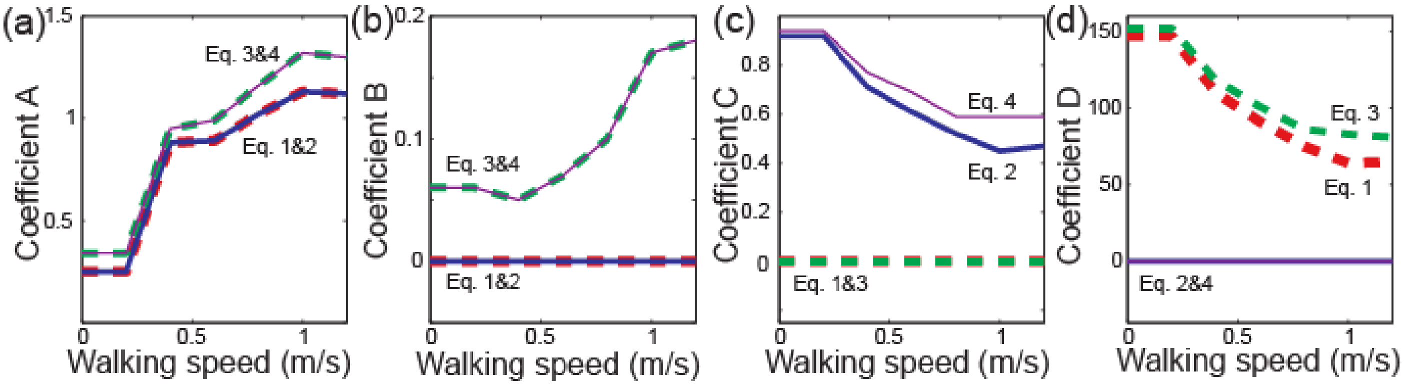

Fig. S1. Force-field coefficient values are illustrated for the four candidate equations, with each coefficient (A-D) corresponding to the panel label. These values vary with walking speed, based on empirically-derived best-fit coefficients from neurologically-intact control participants walking at a range of speeds, and interpolated between these speeds. The four equations are indicated with labels (Eq. 1 through Eq. 4) on each panel.

analyses focus only on the direct effects of the assistance, not the wash-out periods.

C. Force-field Control

This preliminary experiment used the same force-field device described in the main text, and detailed in prior work [3, 9]. For each step, a mechanically-appropriate step width was calculated by one of four Assistive control equations taking the following form:

$$SW = A*x_{pelvis} + B*v_{pelvis} + C*SW_{mean} + D \quad \text{(general Eq.)}$$

Here, x_{pelvis} is the mediolateral location of the sacrum relative to the stance heel at the start of the step. v_{pelvis} is the mediolateral velocity of the sacrum at the start of the step. SW_{mean} is the participant's mean step width, as calculated from the last 50-steps of the initial Normal walking trial. *A*, *B*, *C*, and *D* are coefficients that vary with participant walking speed, as illustrated in Figure S1. Note that for each equation, either the *C* coefficient or *D* coefficient is set to zero, and step width (*SW*) is in units of millimeters.

As an illustrative example, the four equations applied for a participant walking at 0.60 m/s would be:

$$SW = 0.89*x_{pelvis} + 91 \quad \text{(Eq. 1)}$$
$$SW = 0.89*x_{pelvis} + 0.61*SW_{mean} \quad \text{(Eq. 2)}$$
$$SW = 0.99*x_{pelvis} + 0.07*v_{pelvis} + 101 \quad \text{(Eq. 3)}$$
$$SW = 0.99*x_{pelvis} + 0.07*v_{pelvis} + 0.69*SW_{mean} \quad \text{(Eq. 4)}$$

The positive coefficients indicate that for all equations, a larger mediolateral pelvis displacement or velocity away from the stance foot would cause the force-field to encourage a wider step. The equations differ in terms of whether they consider pelvis velocity and each participant's mean step width.

D. Data Collection and Processing

As in the main text, our primary outcome is step start paretic ρ_{disp}, as calculated from LED markers placed on the sacrum and bilateral heels, sampled at 120 Hz, and low-pass filtered at 10 Hz. Secondarily, we also report step start non-paretic ρ_{disp}, and step end ρ_{disp} values for paretic and non-paretic steps.

E. Statistics

Due to the relatively small sample size in this experiment, we used non-parametric statistical comparisons. Specifically, we used a Friedman's test (α=0.05) to compare step start paretic ρ_{disp} values between the baseline Normal walking condition and the four Assistive conditions (corresponding to four control equations). In the case of a significant effect, we used Tukey-Kramer post hoc tests to compare individual conditions. Secondarily, we repeated this analysis for our secondary measures of step start non-paretic ρ_{disp}, step end paretic ρ_{disp}, and step end non-paretic ρ_{disp}.

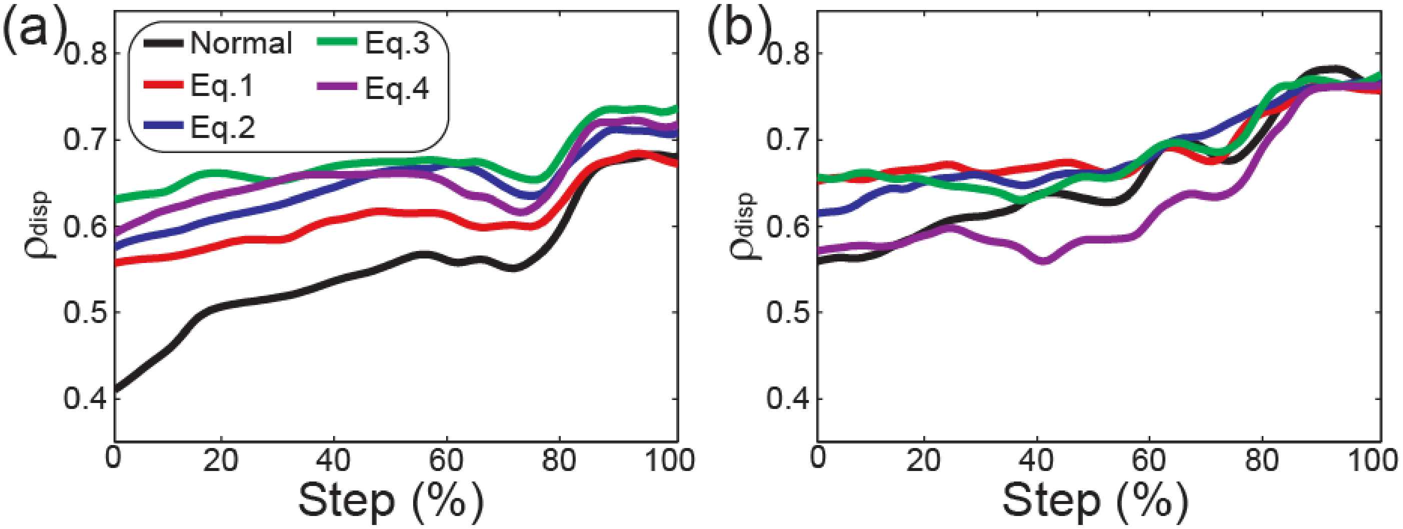

Fig. S2. ρ_{disp} values calculated throughout the step, from step start (0%) to step end (100%). The direct effects and after-effects of each assistance equation are illustrated for steps taken with the paretic leg (a) and the non-paretic leg (b). Plots illustrate the mean value of these metrics across participants.

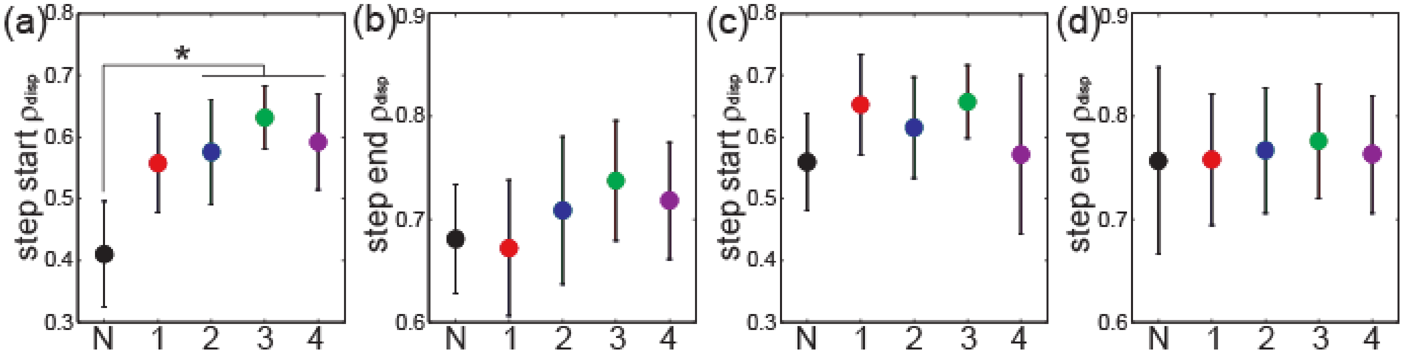

Fig. S3. Force-field effects on ρ_{disp} values. Each panel illustrates a comparison between Normal walking (abbreviated N on x-axis) and the four candidate assistive equations (indicated by numbers 1-4). These comparisons were performed for step start paretic ρ_{disp} (a), step end paretic ρ_{disp} (b); step start non-paretic ρ_{disp} (c); and step end non-paretic ρ_{disp} (d). Dots indicate group means, and error bars indicate 95% confidence intervals. The asterisk indicates a statistically significant post-hoc difference between conditions.

III. Results

As observed in the main text, the partial correlation between pelvis displacement and step width (ρ_{disp}) increased over the course of a step, and was generally lower for paretic steps (Fig. S2a) than for non-paretic steps (Fig. S2b).

For paretic steps, step start ρ_{disp} varied significantly (p=0.004; Fig. S3a) across the five conditions. Each of the assistance equations other than Equation 1 caused a significant increase in step start paretic ρ_{disp}, and no significant differences were observed between equations. Step end paretic ρ_{disp} did not differ significantly (p=0.082; Fig. S3b) across the five walking conditions. For non-paretic steps, no significant differences across the five walking conditions were observed for step start ρ_{disp} (p=0.068; Fig. S3c) or step end ρ_{disp} (p=0.87; Fig. S3d).

IV. Discussion

For our primary metric (step start paretic ρ_{disp}), we observed no significant differences across the four candidate equations. This result is consistent with the previously observed primary role of pelvis displacement in predicting step width, and only a secondary role of other biomechanical factors [6]. Equations 2, 3, and 4 all produced significant increases in step start paretic ρ_{disp} relative to the baseline walking condition. Based on these results, we chose to apply Equation 2 in this study's main experiment. This choice was based on our ultimate goal of developing a tool that can be implemented in a clinical setting. Equation 2 involves only displacement-dependent forces, which could be feasibly implemented using a passive system of pulleys and springs (which can be simplistically thought of as converting displacements to forces). The inclusion of a velocity term in Equations 3 and 4 would require a more complex design, possibly involving damping (or negative damping) elements.

For Equation 2, we calculated an effect size of 1.2 for our primary outcome measure of step start paretic ρ_{disp}, as assistance caused an increase in this measure of 0.17±0.14 (mean±s.d.). Given this effect size, we calculated that a sample size of 10 would be required to achieve 80% power with an alpha value of 0.025 – as planned for our main experiment.

Appendix B

I. Introduction

Our focus in the main text was on the partial correlation between mediolateral pelvis displacement and step width. This choice was based on our prior finding that this relationship is significantly weakened for paretic steps among PwCS [1]. In contrast, we did not observe a significant difference between paretic and non-paretic steps in terms of the partial correlation between mediolateral pelvis velocity and step width [1], suggesting that our focus on pelvis displacement is warranted. However, several prior studies (including some of our own) have quantified the relationship between pelvis motion and step width using the R^2 magnitude calculated from linear regressions that included both pelvis displacement and velocity as independent variables [5-6, 10-11]. Therefore, for comparison purposes we here provide a secondary analysis of the effects of walking in our force-field in terms of this R^2 metric, as well as the partial correlation between mediolateral pelvis velocity and step width.

II. Methods

The presented analyses correspond to the 20 participants described in detail in the main text.

For steps taken with the paretic leg, we performed a linear regression with the dependent variable of step width, and the independent variables of mediolateral pelvis displacement and velocity at the start of the step. The magnitude of the R^2 value from this regression (step start paretic R^2) is interpreted as the proportion of the variability in step width that can be predicted from pelvis motion at the start of the step. We repeated the same analysis to predict paretic step width from mediolateral pelvis displacement and velocity at the end of the step (step end paretic R^2). These two regressions were then performed for steps taken with the non-paretic leg (producing metrics of step start non-paretic R^2 and step end non-paretic R^2).

For steps taken with the paretic leg, we also calculated the partial correlation between step width and mediolateral pelvis velocity at the start of the step (step start paretic ρ_{vel}), accounting for the mediolateral pelvis displacement at the start

of the step. Following the same pattern described above, the corresponding metric was calculated based on pelvis velocity at the end of the step (step end paretic ρ_{vel}), and at both time points for steps taken with the non-paretic leg (step start non-paretic ρ_{vel} and step end non-paretic ρ_{vel}).

For each of the aforementioned metrics, we used Wilcoxon signed rank tests to compare walking periods of interest. Just as in the main text, for the Assistive group, we compared the Normal and Assistive periods (direct effects) and the Normal and Transparent periods (after-effects). For the Perturbing group, we compared the Normal and Perturbing periods (direct effects) and the Normal and Transparent periods (after-effects). P-values of less than 0.025 were interpreted as significant.

III. Results

Force-field assistance did not significantly influence paretic stepping behavior quantified with R^2 metrics, paralleling the

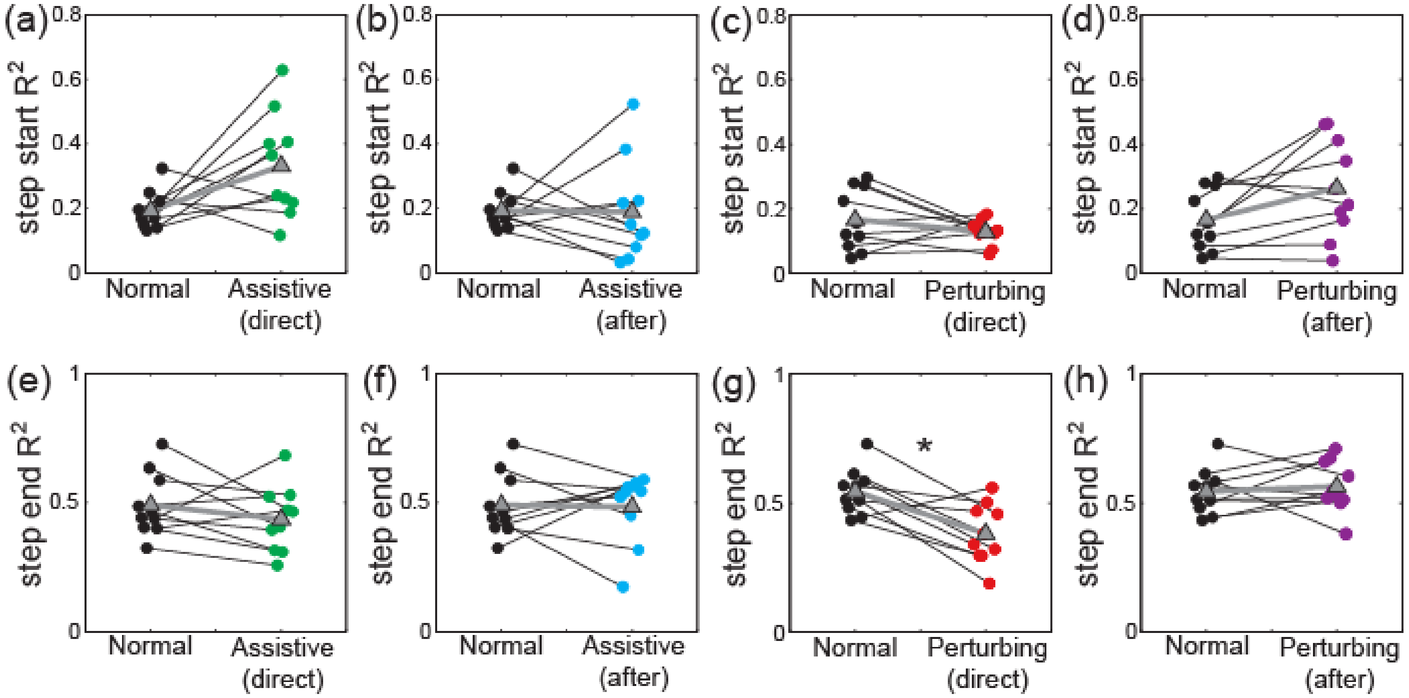

Fig. S4. Force-field effects on paretic R^2. All panels illustrate individual participants' values for these metrics, and the change relative to Normal walking. The top row (panels a-d) illustrates step start paretic R^2, while the bottom row (panels e-h) illustrates step end paretic R^2. From left to right, the panels in each row illustrate the direct effects of assistance, the after-effects of assistance, the direct effects of perturbations, and the after-effects of perturbations. Small circles and thin black lines indicate individual values, outlined triangles and thick gray lines indicate group means, and statistical significance is illustrated with asterisks (*). Individual data points are slightly offset on the x-axis to reduce overlap.

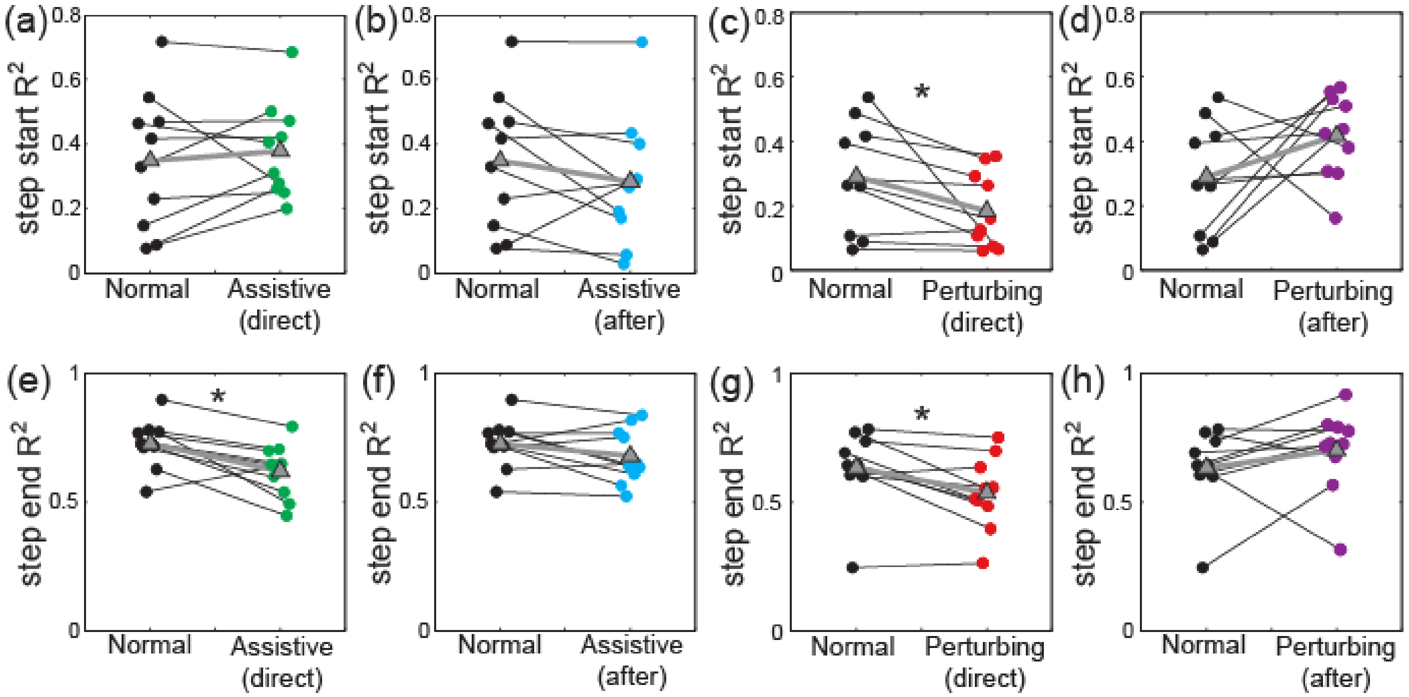

Fig. S5. Force-field effects on non-paretic R^2. The figure follows the same structure as Fig. S4.

main text results with paretic ρ_{disp}. Specifically, assistance did not have a significant direct effect (p=0.065; Fig. S4a) or after-effect (p=0.63; Fig. S4b) on step start paretic R^2. Similarly, assistance did not have a significant direct effect (p=0.23; Fig. S4e) or after-effect (p=0.92; Fig. S4f) on step end paretic R^2. Force-field perturbations had less notable effects on paretic R^2 than those observed in the main text with paretic ρ_{disp}. Perturbations did not have a significant direct effect (p=0.32; Fig. S4c) or after-effect (p=0.11; Fig. S4d) on step start paretic R^2. Perturbations had the significant direct effect of decreasing step end paretic R^2 (p=0.010; Fig. S4g), but had no significant after-effect on this metric (p=0.70; Fig. S4h).

For non-paretic steps, force-field assistance had no significant direct effect (p=0.43; Fig. S5a) or after-effect (p=0.19; Fig. S5b) on step start R^2. Unexpectedly, assistance had the direct effect of significantly decreasing step end non-paretic R^2 (p=0.020; Fig. S5e), but had no significant after-effect on this metric (p=0.19; Fig. S5f). Perturbations had the significant direct effect of decreasing both step start non-paretic R^2 (p=0.010; Fig. S5c) and step end non-paretic R^2 (p=0.010; Fig. S5g), but did not have a significant after effect on either

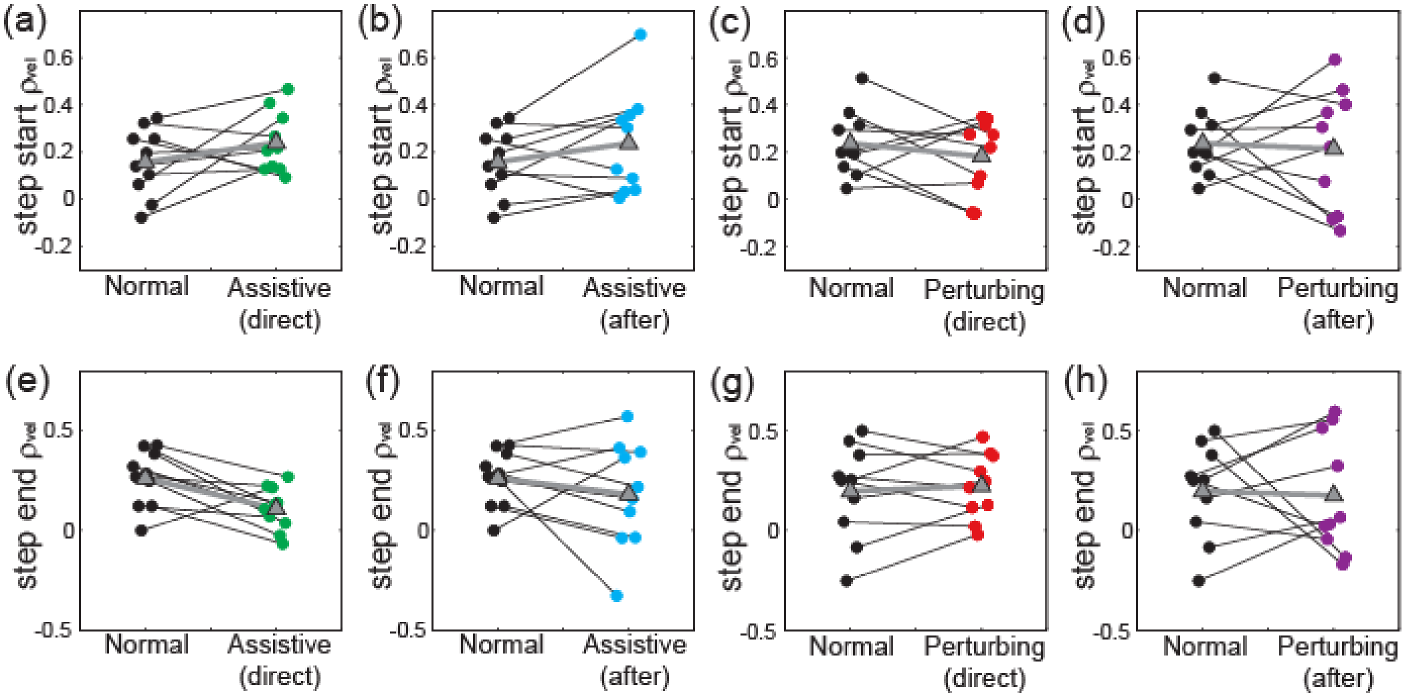

Fig. S6. Force-field effects on paretic ρ_{vel}. The figure follows the same structure as Fig. S4.

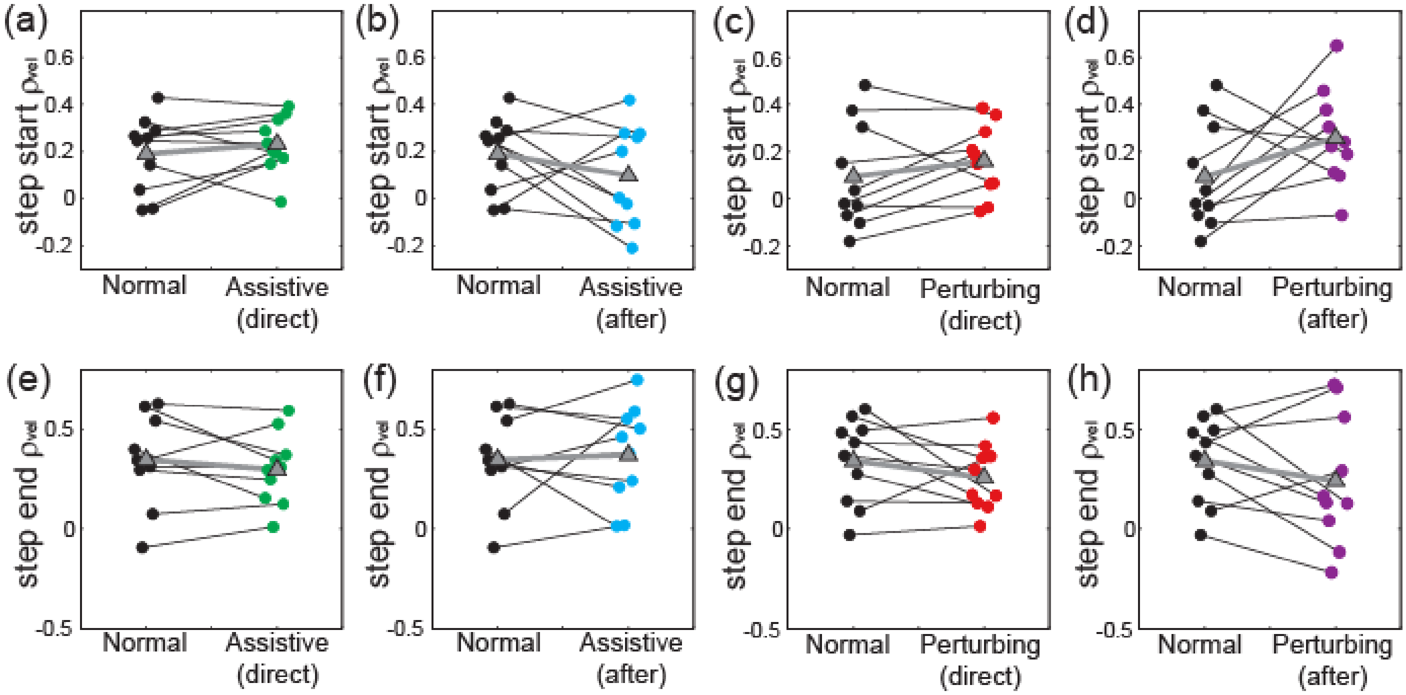

Fig. S7. Force-field effects on non-paretic ρ_{vel}. The figure follows the same structure as Fig. S4.

step start non-paretic R^2 (p=0.13; Fig. S5d) or step end non-paretic R^2 (p=0.16; Fig. S5h).

Unlike with either ρ_{disp} or R^2 magnitude, we observed no significant changes in ρ_{vel} across any comparisons. Individual ρ_{vel} data are illustrated in Fig. S6 for paretic steps, including the direct effects of assistance on step start ρ_{vel} (p=0.28; Fig. S6a), the after-effects of assistance on step start ρ_{vel} (p=0.28; Fig. S6b), the direct effects of perturbations on step start ρ_{vel} (p=0.38; Fig. S6c), the after-effects of perturbations on step start ρ_{vel} (p=0.85; Fig. S6d), the direct effects of assistance on step end ρ_{vel} (p=0.037; Fig. S6e), the after-effects of assistance on step end ρ_{vel} (p=0.28; Fig. S6f), the direct effects of perturbations on step end ρ_{vel} (p=0.70; Fig. S6g), and the after-effects of perturbations on step end ρ_{vel} (p=0.85; Fig. S6h). Individual ρ_{vel} data are illustrated in Fig. S7 for non-paretic steps, including the direct effects of assistance on step start ρ_{vel} (p=0.43; Fig. S7a), the after-effects of assistance on step start ρ_{vel} (p=0.28; Fig. S7b), the direct effects of perturbations on step start ρ_{vel} (p=0.19; Fig. S7c), the after-effects of perturbations on step start ρ_{vel} (p=0.11; Fig. S7d), the direct effects of assistance on step end ρ_{vel} (p=0.43; Fig. S7e), the after-effects of assistance on step end ρ_{vel} (p=0.85; Fig. S7f), the direct effects of perturbations on step end ρ_{vel} (p=0.23; Fig. S7g), and the after-effects of perturbations on step end ρ_{vel} (p=0.28; Fig. S7h).

IV. Discussion

In our previous work, we found changes in the investigated R^2 metric to largely parallel changes in ρ_{disp}. Specifically, among PwCS, steps taken with the paretic leg had lower R^2 values and ρ_{disp} values than steps taken with the non-paretic leg, whereas ρ_{vel} values did not differ between legs [1]. Among neurologically-intact controls, changes in walking speed had similar effects on the patterns of both R^2 and ρ_{disp} values throughout a step, while ρ_{vel} differed substantially [6]. Finally, delivering assistance or perturbations to neurologically-intact controls using our novel force-field caused similar changes in the R^2 and ρ_{disp} metrics [9, 12].

In the present work, the changes in R^2 were generally similar to the changes in ρ_{disp}, with a few exceptions. Most notably, force-field perturbations during paretic steps had the direct effect of significantly reducing step start ρ_{disp}, but did not significantly affect step start R^2. We attribute this difference to the nature of the R^2 metric. For several participants, perturbations reduced paretic step start ρ_{disp} to below zero, as larger pelvis displacements were followed by narrower steps. As R^2 values cannot be negative (a floor effect), the magnitude of possible decreases was limited, consistent with the tight clustering in Fig. S4c. Based on this observation, we believe that ρ_{disp} is a more appropriate metric than R^2 to detect the full range of behaviors observed in PwCS. We also found force-field perturbations during paretic steps to produce significant positive after-effects in ρ_{disp}, but not R^2. The explanation for this discrepancy is not as straightforward. Two participants exhibited positive after-effects in ρ_{disp} but not R^2 due to substantial decreases in the magnitude of ρ_{vel}. Given the lack of consistent changes in ρ_{vel} (discussed briefly below), it is unclear whether this should be interpreted as important. Finally, we found that both assistance and perturbations had the direct effect of causing significant decreases in non-paretic step end R^2 (p=0.020 and p=0.010, respectively). While significant decreases were not observed in non-paretic step end ρ_{disp}, we did see non-significant trends in the same direction (p=0.037 and p=0.028, respectively), suggesting that these results would likely have been consistent with a larger sample size.

Unlike with both ρ_{disp} and R^2, force-field assistance and perturbations did not cause significant changes in ρ_{vel}. This result is likely due to the control equation in the main text not including pelvis velocity as an input variable. With this type of a control equation, previous work in neurologically-intact controls similarly found no effect of assistance or perturbations on ρ_{disp} [9].

References

[1] K. H. Stimpson, L. N. Heitkamp, A. E. Embry, J. C. Dean, "Post-stroke deficits in the step-by-step control of paretic step width," *Gait Posture*, vol. 70, p. 136-140, 2019.

[2] S. M. Bruijn, J. H. van Dieen, "Control of human gait stability through foot placement," *J R Soc Interface*, vol. 15, p. 20170816, 2018.

[3] E. T. Nyberg, J. Broadway, C. Finetto, J. C. Dean, "A novel elastic force-field to influence mediolateral foot placement during walking," *IEEE Trans Neur Syst Rehabil Eng*, vol. 25, p. 1481-1488, 2017.

[4] B. L. Rankin, S. K. Buffo, J. C. Dean, "A neuromechanical strategy for mediolateral foot placement in walking humans," *J Neurophysiol*, vol. 112, p. 374-383, 2014.

[5] Y. Wang, M. Srinivasan, "Stepping in the direction of the fall: the next foot placement can be predicted from current upper body state in steady-state walking," *Biol Lett*, vol. 10, p. 20140405, 2014.

[6] K. H. Stimpson, L. N. Heitkamp, J. S. Horne, J. C. Dean, "Effects of walking speed on the step-by-step control of step width," *J Biomech*, vol. 68, p. 78-83, 2018.

[7] R. C. Browning, R. Kram, "Effects of obesity on the biomechanics of walking at different speeds," *Med Sci Sport Exerc*, vol. 39, p. 1632-1641, 2007.

[8] L. Hak, H. Houdijk, F. Steenbrink, A. Mert, P. van der Wurff, P. J. Beek, J. H. van Dieen, "Speeding up or slowing down? Gait adaptations to preserve gait stability in response to balance perturbations," *Gait Posture*, vol. 36, p. 260-264, 2012.

[9] L. N. Heitkamp, K. H. Stimpson, J. C. Dean, "Application of a novel force-field to manipulate the relationship between pelvis motion and step width in human walking," *IEEE Trans Neur Syst Rehabil Eng*, vol. 27, p. 2051-2058, 2019.

[10] M. Mahaki, S. M. Bruijn, J. H. van Dieen, "The effect of lateral stabilization on the use of foot placement to control mediolateral stability in walking and running," *PeerJ*, vol. 7, p. e7939, 2019.

[11] M. Wu, G. L. Brown, J. L. Woodward, S. M. Bruijn, K. E. Gordon, "A novel Movement Amplification environment reveals effects of controlling lateral centre of mass motion on gait stability and metabolic cost," *R Soc Open Sci*, vol. 7, p. 198009, 2020.

[12] N. K. Reimold, H. A. Knapp, R. E. Henderson, L. Wilson, A. N. Chesnutt, J. C. Dean, "Altered active control of step width in response to mediolateral leg perturbations while walking," *Sci Rep*, vol. 10, p. 12197, 2020.
